# Opportunities and challenges in the current era of global medical education

**DOI:** 10.5116/ijme.5ad1.ce9a

**Published:** 2018-04-27

**Authors:** Muhammad Rizwan, Nicole Rosson, Sean Tackett, Heitham Hassoun

**Affiliations:** 1 Johns Hopkins University School of Medicine and Johns Hopkins Medicine International. Baltimore, USA

**Keywords:** Opportunities, challenges, current era, global medical education, USA

## Introduction

The global healthcare market is massive and expanding and is having an unprecedented influence on medical education around the world.^1^ Increased demand for healthcare has created demand for physicians over and above the global shortage of physicians that has been well-recognized. This heightened demand for physicians has led to a number of trends, such as an exponential increase in the number of medical schools and medical students and migration for medical education and training.^2^^-^^6^ This new global medical education system, marked by its growing size and complexity, has led to greater concerns about quality assurance of individual graduates and their educational programs. The purpose of this article is to describe current trends in international medical education and how this has motivated others to act to assure the quality of individual graduates and educational programs.

### International medical schools and migration of medical students

The number of medical schools around the world has been increasing dramatically over the last several decades, particularly in emerging economies, in response to legacies of physician shortages and the increased demand for healthcare. In some locations, such as India, Pakistan, China, and Brazil, this rapid growth is potentially beneficial to scaling up physician training and meeting population needs.  However, in other locations, notably the Caribbean, there are far more medical schools than are needed to serve the local population. This asymmetric growth in medical schools is likely fostered by an increased willingness of individuals to travel for their medical education.  While the “brain drain” of trained physicians from low income to high-income settings has been well-recognized, migration for undergraduate medical education is a growing trend.  Medical education programs that are taught entirely in English have developed in non-English speaking countries, including those in Eastern Europe, Russia, Ukraine, and China, to attract international students and allow graduates greater mobility across European borders and entrance to practice in English-speaking areas.^4^^-^^9^ With the language barrier removed, students often seek these international medical schools as admission may be less competitive or tuition costs lower than schools in their home countries.  Additionally, some schools, such as several in the Caribbean, have modeled and developed their admissions processes and curricula after US medical schools to attract international students.^10^

These different driving forces - the urge to seek medical education at lower cost and at institutions that have less competitive admissions processes have ushered in more complex patterns of migration than those of traditional “brain drain”. Generally, there has been a decrease in the number of international students opting to study in “resource-rich” countries,^2^^,^^4^ although many students still migrate from areas where medical education may not be possible.  Currently, North America, South Asia, and Africa are the largest sending regions, and the Americas, Eastern Europe, China, and Russia are the most common receiving regions of international medical students worldwide.^3^^,^^5^^,^^6^^,^^10^ China provides an example of how student flows are taking new patterns.  There, health professional students are currently the third largest group among all international students, with the largest influxes coming from South Asian and African countries.^9^

### Quality assurance of individuals and educational programs

The increases in new and non-traditional medical schools and medical student migration have been drawing greater attention to assuring the quality of individual graduates and medical education programs.^11^^,^^12^ Many new medical schools are starting in areas with limited quality assurance mechanisms, leading to concerns about for-profit and predatory schools that capitalize on a student’s desire to become a physician and on their ability to pay tuition.  For example, reports of some private medical schools have drawn attention to their lack of formal testing or exams, and unconventional practices for granting credit hours.^13^

How quality is assured in medical education varies considerably from country to country, as do training models. However, traditionally there have been two ways to assure quality, assessment of the individual practitioner (e.g., licensure examinations) and accreditation of a school or educational program. The globalization of the medical workforce and evidence that suggests that foreign medical graduates perform more poorly on standardized exams than graduates from local schools is leading to interest in more uniform ways to conduct each quality assurance process.^14^ The National Board of Medical Examiners, which administers the series of licensing exams in the US has partnered with international groups for decades and has created the International Foundation of Medicine exams, which are being increasingly used for assessment throughout the world. Some have advocated for uniform assessments and licensure processes that allow individuals to cross borders to practice medicine and would create a genuinely global physician workforce.  Still, whether a country should have a national licensing exam is hotly debated due to limitations in the evidence available to show that these exams improve clinical practice and protect public safety.

Driven primarily by the rapid growth in schools, accreditation of medical education programs or schools is drawing considerable interest from the international community. The first set of global standards was published by the World Federation for Medical Education (WFME) in 2003.^15^ In 2010, the Educational Commission for Foreign Medical Graduates announced that by 2023 all applicants would need to have graduated from a program accredited by an authority that met WFME or other global criteria for an accrediting body.^16^ Since then accreditation for all health training programs by 2020 was recommended as part of the World Health Organization’s Global Strategy on Human Resources for Health: Workforce 2030 and was endorsed by the World Medical Association.^17^ While it seems unlikely that all areas with medical schools will have a WFME-recognized accrediting authority by 2023, that a growing number of agencies are applying for recognition, and completing the recognition process, suggests that the enthusiasm of international organizations for accreditation is being taken up by local authorities. Indeed, the growth in medical schools shows no signs of slowing, so that some mechanism of formal external peer review of medical schools is likely to benefit medical education internationally.

## Conclusions

Globalization has dramatically impacted medical education and the development of physicians, and the international landscape is likely to become ever more complex. Recognizing the emerging trends in medical school growth and student migration will be critical to directing the evolution of regulatory and quality assurance mechanisms to ensure all physicians received a high-quality education that will permit the medical education community to meet population health needs and maintain public trust.

### Conflict of Interest

The authors declare that they have no conflict of interest.
